# Multi‐angle fluoroscopic lung perfusion imaging using x‐ray pulsatility index: A pilot study in healthy subjects

**DOI:** 10.1002/mp.70666

**Published:** 2026-09-02

**Authors:** Matthew R. Smith, Bradley W. Richmond, Savannah Gregory, Maggie Cartwright, Andreas Fouras, Greg T. Mogel, Charles R. Hatt, Gary T. Smith, Jared V. Grice

**Affiliations:** ^1^ Department of Radiology University of Wisconsin School of Medicine and Public Health Madison Wisconsin USA; ^2^ Department of Veterans Affairs Medical Center Nashville Tennessee USA; ^3^ Division of Allergy, Pulmonary, and Critical Care Medicine Vanderbilt University School of Medicine Nashville Tennessee USA; ^4^ Department of Cell and Developmental Biology Vanderbilt University Nashville Tennessee USA; ^5^ Department of Medicine, Division of Allergy, Pulmonary, and Critical Care Medicine Vanderbilt Health Nashville Tennessee USA; ^6^ Vanderbilt University Institute of Imaging Science Nashville Tennessee USA; ^7^ 4D Medical Melbourne Australia; ^8^ Department of Radiology Vanderbilt Health Nashville Tennessee USA; ^9^ Vanderbilt University Institute for Imaging Science Vanderbilt Health Nashville Tennessee USA

**Keywords:** lung perfusion, spectral analysis, x‐ray pulsatility index

## Abstract

**Background:**

X‐ray pulsatility index (XPI) quantifies cardiac‐synchronous attenuation changes as a surrogate for regional lung perfusion, but current implementations are limited by projectional overlap and the lack of reference values in healthy individuals.

**Purpose:**

To characterize regional XPI patterns and establish preliminary reference values in healthy participants using a novel multi‐angle fluoroscopic imaging system.

**Methods:**

Nineteen healthy participants underwent an 8 s breath‐hold during fluoroscopic imaging using 15 fps at four simultaneous projections (LPO, AP caudal, RPO, AP cranial) using a novel scanner. Frames were cropped to exclude initial x‐ray tube stabilization and bulk motion. Spectral analysis exploited the periodic signal attenuation in the lungs to create XPI maps, as previously described. To focus on the clinically important regions of lung perfusion, the peripheral 3 cm of lung was manually segmented and divided into upper, middle, and lower lung zones. XPI values from these regions were compared to evaluate laterality and cranial–caudal differences using paired *t*‐tests corrected with the Benjamini‐Hochberg procedure to control the False Discovery Rate (FDR). Peripheral XPI contrast‐to‐noise (pCNR) was calculated in the AP caudal projection to quantify signal‐to‐background separation. Data were retrospectively resampled to assess the effect of reduced frame rate (7.5, 5 fps) and shorter acquisition time using multiple regression analysis. Radiation dose was estimated using phantom measurements and simulation.

**Results:**

All participants performed the breath‐hold without difficulty. XPI maps demonstrated bilateral lung perfusion across all four views, enabling multi‐projection assessment of regional perfusion. No focal defects were observed; however, one participant demonstrated globally reduced XPI. Significant cranial–caudal gradients in XPI were observed across projection angles, consistent with known gravity‐dependent physiology. Following FDR adjustment (*Q* = 0.05), the lower zones continued to demonstrate significantly higher values than the upper zones in 3 of 4 projections (all *q* < 0.05). There were no significant laterality differences (*q* > 0.05). XPI values remained stable across acquisition lengths and frame rates, indicating robustness of the metric. CNR increased with longer acquisitions and higher frame rates, reflecting reduced noise and improved signal reliability.

**Conclusion:**

Multi‐angle XPI fluoroscopy enables non‐invasive regional lung perfusion assessment with low radiation exposure and provides preliminary reference values for future clinical investigation.

## 1 INTRODUCTION

Accurate, non‐invasive assessment of regional lung perfusion is critical for understanding pulmonary physiology and detecting early disease, yet current imaging options remain limited. Conventional modalities such as nuclear medicine ventilation–perfusion scans or dual‐energy CT require intravenous contrast or prolonged acquisition times, which can restrict their clinical use. Recent advances in fluoroscopic technology^1^ offer the opportunity to extract physiologic signals from routine x‐ray data with substantially lower dose and without contrast administration.

Prior studies have shown that dynamic chest radiography (DCR)‐derived signal changes correlate with pulmonary perfusion and can detect regional abnormalities.^2^, ^3^, ^4^, ^5^, ^6^, ^7^, ^8^, ^9^, ^10^ However, the extent to which these signals specifically reflect perfusion versus combined cardiopulmonary motion remains an area of ongoing investigation. Central lung measurements are particularly vulnerable to projectional overlap and contamination from bulk cardiac motion, potentially reducing physiologic specificity. These approaches predominantly emphasize central signal in their display and often exclude peripheral or extra‐pulmonary regions in their analysis, limiting robust signal‐to‐background assessment. Although prior studies have demonstrated feasibility for detecting perfusion defects, normative peripheral‐lung reference values remain undefined. As a result, characterization of peripheral perfusion—where projectional confounders are reduced—remains an important and underexplored area. In contrast to prior DCR approaches that focus quantitative measurements on centrally derived signal, the present study specifically focuses quantitation on peripheral lung tissue, enabling evaluation in regions less affected by cardiac motion and projectional overlap.

The x‐ray pulsatility index (XPI) is an alternative method to isolate subtle cardiac‐related oscillations in lung tissue attenuation using Fourier‐based band‐pass filtering. This method is sensitive to low‐level changes in signal attenuation, as demonstrated in phantom models,^11^ and as a surrogate of perfusion in preliminary studies with acute pulmonary artery occlusions^12^ and chronic thromboembolic pulmonary hypertension (CTEPH).^13^ Establishing XPI values in a normal population and understanding the influence of acquisition parameters are essential next steps toward clinical translation. In this prospective pilot study, we evaluated a multi‐angle fluoroscopic system in upright healthy participants to define baseline XPI measurements, assess the feasibility of dose‐reduction strategies, and explore physiologic perfusion patterns across lung regions.

We hypothesized that XPI would demonstrate (1) no significant laterality differences and (2) a gravity‐dependent cranial–caudal gradient in healthy subjects seated in the upright position. We further hypothesized that XPI values would remain robust to shorter scan times and lower frame rates.

## 2 METHODS

This prospective study was approved by the institutional review board (#241021) and all participants provided written consent. Healthy asymptomatic participants without a history of known pulmonary disease were recruited from the general community. Participants were excluded if respiratory medications were used and if they had any history of cardiovascular disease.

### 2.1 Image acquisition and XPI processing

**FIGURE 1:**
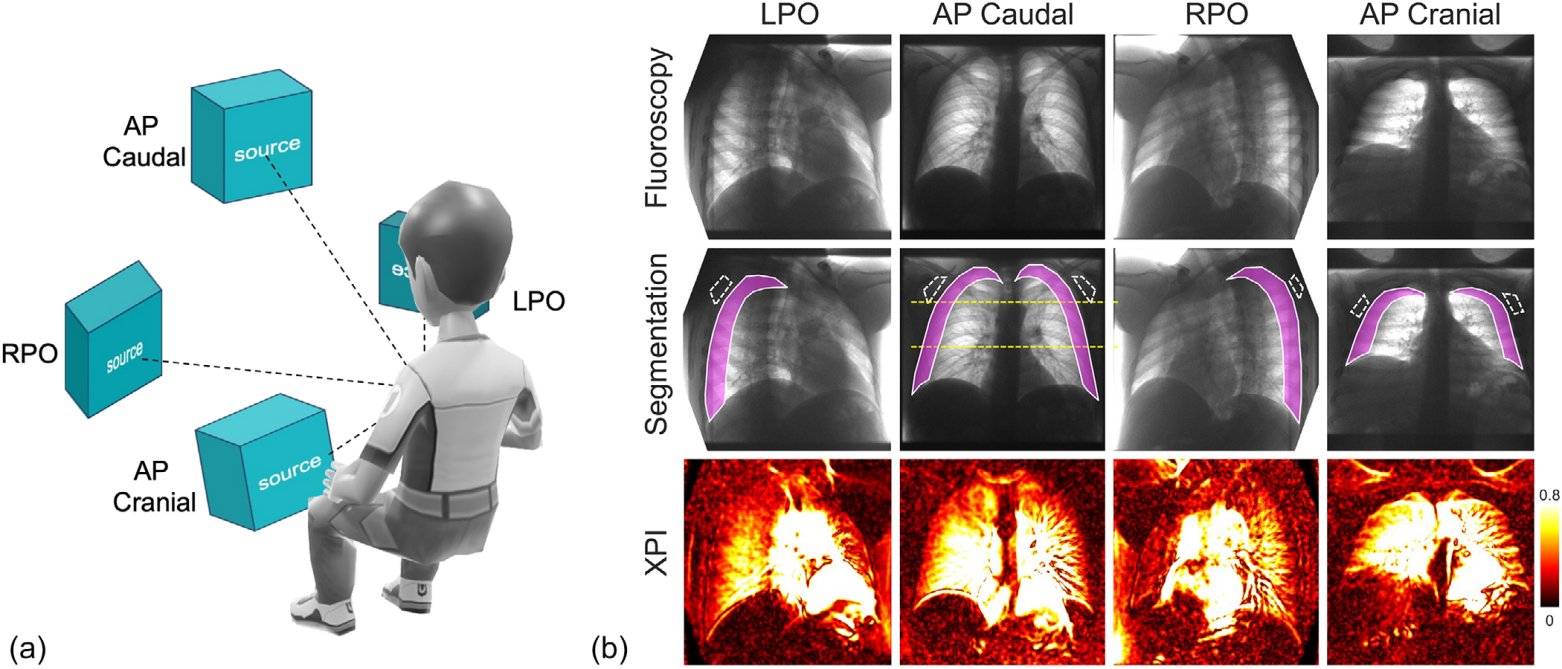
(a) Novel fluoroscopic system acquires four projection angles simultaneously with participants in an upright seated position. (b) Example fluoroscopy views (top row), manual segmentation examples of the peripheral 3 cm of tissue and background regions‐of‐interest (middle row), and corresponding XPI maps (bottom row), highlighting the multi‐angle evaluation of the lungs. Noise was measured in background regions‐of‐interest in the ipsilateral side of the investigated lung (i.e., right shoulder for right lung). The right shoulder was used for noise measurements for the two AP views. LPO and RPO elongate the right and left lungs respectively. AP caudal inlay shows example of vertical zonal distribution.

Subjects were seated upright in a multi‐angle fluoroscopic scanner (XV scanner, 4DMedical, Melbourne, Australia) equipped with four unique projection angles (−22.5° LPO, 55° AP caudal, 22.5° RPO, −55° AP cranial per manufacturer specifications) as shown in Figure 1a. Each participant was coached on breathing and performed an 8 s breath hold following inspiration. Fluoroscopy was acquired at 15 fps, 10 ms pulses with tube settings adjusted by the automatic exposure control unit. The dynamic images were downloaded for offline processing.

Each acquisition was processed using techniques previously described.^11^ First, the set of contiguous frames without diaphragm motion were identified and temporal signal normalized pixel‐by‐pixel. The temporal signal from each pixel was converted into the frequency domain using the Fourier Transform. The band‐pass filter was centered at the dominant peak in the signal spectrum corresponding to the heart rate using a bandwidth of 10 bpm to capture the desired signal and minimize noise. This filter was applied to each voxel in the image which isolates the component of the signal related to blood‐flow. The magnitude of this oscillation, termed x‐ray pulsatility index (XPI), is displayed as a map following Gaussian smoothing with standard deviation of 7 pixels (Figure 1b).

### 2.2 Acquisition length and frame rate analysis

To assess robustness to shorter scan times, temporal frames for each participant were iteratively removed from the end of the acquisition, and the resulting contiguous subset of frames were processed into XPI maps. The minimum total acquisition time was set to 1.5 s. To determine the effect of frame rate, each original acquisition (15 fps) was downsampled in the temporal dimension to simulate additional varying frame rates (7.5, 5 fps). For example, an acquisition with 7.5 fps was reconstructed using every other timepoint, and 5 fps was reconstructed using every 3^rd^ timepoint (i.e. removing frames rather than interpolation). This method ignored the temporal averaging that often occurs with clinical fluoroscopy and thus yielded a representation of different frame rates rather than an independent acquisition.

### 2.3 Peripheral lung signal

Central vascular structures have strong projectional overlap on XPI maps and the central lung is also confounded by bulk cardiac motion. Therefore, to avoid these regions of bias, the peripheral 3 cm of lung was manually segmented on the fluoroscopic image (Figure 1b) by M.S. blinded to XPI and further divided into thirds for upper, middle, and lower lung zones. Pairwise comparisons between lung zones (upper, middle, lower) were performed within each projection to evaluate cranial–caudal perfusion differences. Lateral differences between the right and left lungs were evaluated using the LPO and RPO projections respectively. In total, 21 hypothesis tests were performed (Table S1). To control for multiple comparisons, the Benjamini–Hochberg false discovery rate procedure was applied with a false discovery rate threshold *Q* = 0.05, and FDR‐adjusted *q*‐values are reported; comparisons were considered significant if *q* < 0.05. Multiple regression analysis was used to evaluate the resampled data to assess the effect of reduced frame rate and shorter acquisition time.

Because XPI represents a relatively small percent signal change over the cardiac cycle, adequate signal‐to‐background separation is important for establishing technical robustness. Contrast‐to‐noise ratio (CNR) provides a quantitative measure of this separation. The peripheral XPI contrast‐to‐noise (pCNR) was calculated using $pCNR=\left({\bar{XPI}}_{Lung}-\;{\bar{XPI}}_{Bkg}\right)/std\left(XPI_{Bkg}\right)$ where ${\bar{XPI}}_{Lung}$ is the mean XPI signal in the segmented peripheral lung and $XPI_{Bkg}$ is the signal in a background region of interest, which was placed over the shoulder outside the lung in an area containing homogenous signal (Figure 1). pCNR measurements for the AP views (cranial, caudal) used background from the right shoulder, while pCNR measurements for the oblique views (LPO, RPO) used the background from the right and left shoulder respectively. The pCNR was plotted as a function of acquisition length and frame rate.

### 2.4 Radiation dose estimation

The radiation dose from the x‐ray system was estimated by measuring the output from each of the four x‐ray tubes independently with a solid‐state sensor (RadCal Corp., Monrovia, CA) calibrated within the year. Exposures were taken from each tube independently with an anthropomorphic chest phantom (Kyoto Kaguku, Kyoto, Japan) in the field of view at known source‐to‐chamber distances. The scout image and dynamic acquisition were summed to measure cumulative exposure. Source‐to‐skin distances (SSD) for the anthropomorphic phantom were measured and used to calculate entrance skin exposure for each x‐ray tube. The entrance skin exposure, beam quality, and tube geometry were input into a radiation dose estimation software, PCXMC,^14^ to calculate the effective dose for the maximum 13 s exposure used with the phantom, which was then scaled linearly to the 8 s exposure length used in the human study.

## 3 RESULTS

Nineteen participants (3 female, 16 male) were enrolled and performed the breath‐hold without difficulty. Mean age and BMI was 37.0 ± 9.9 years and 26.6 ± 2.9 kg/m^2^ respectively. A single participant demonstrated excessive motion with less than 1.5 s of motion‐free contiguous frames and was thus excluded from all further analyses. Therefore, 18 participants were subsequently analyzed.

**FIGURE 2:**
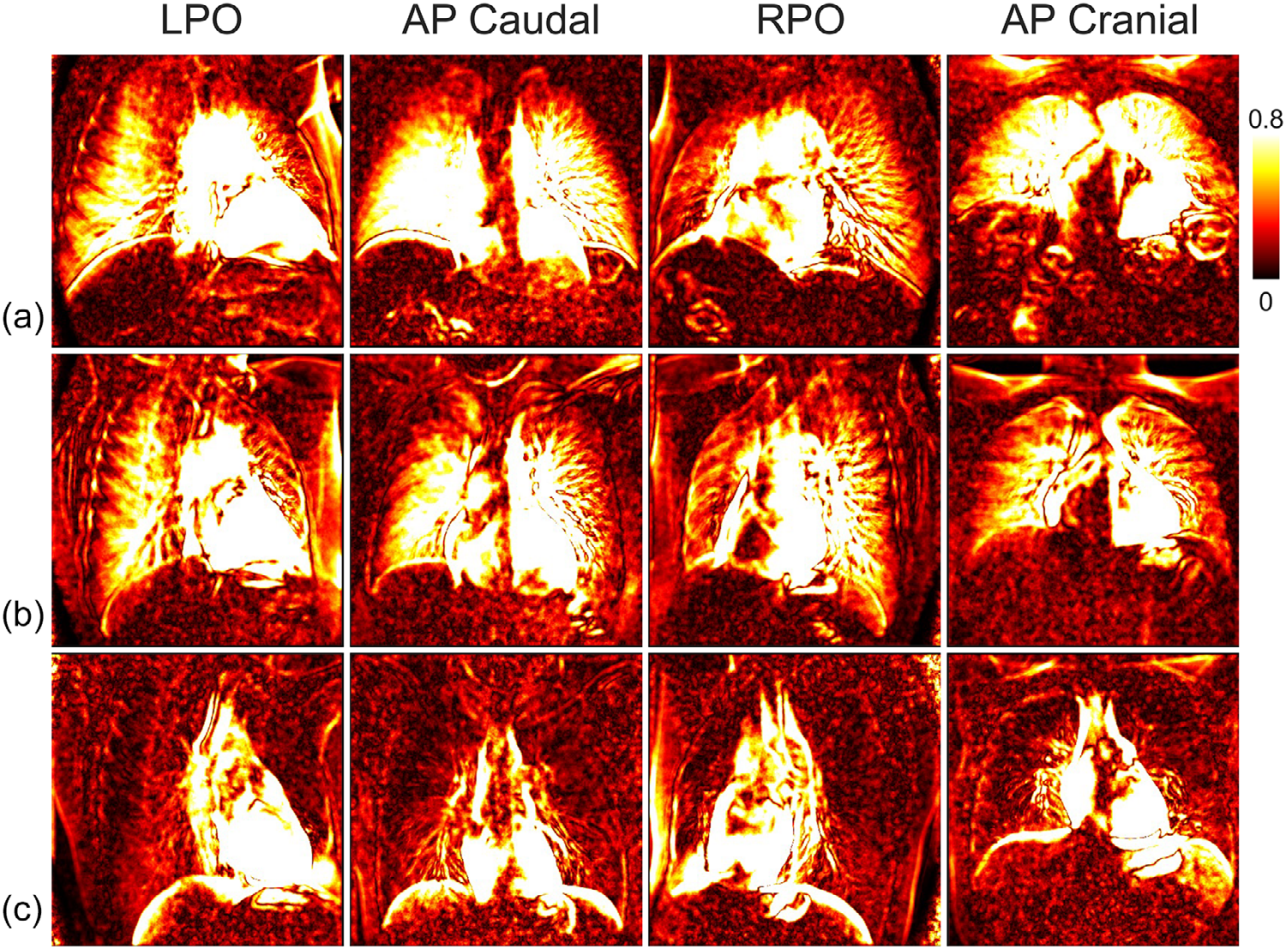
Representative multi‐angle x‐ray pulsatility index (XPI) maps from three participants. Projection views include left posterior oblique (LPO), anteroposterior (AP) caudal, right posterior oblique (RPO), and AP cranial. The oblique projections improve three‐dimensional assessment of the posterior right and left lungs, respectively. (a,b) Participants demonstrating characteristic superior‐to‐inferior gradients in XPI signal intensity. (c) Participant with minimal detectable intrapulmonary XPI signal despite technically adequate image acquisition; this individual was subsequently noted to have a low VO_2_ max, a clinical measure of cardiorespiratory fitness.

The four simultaneous fluoroscopic views enabled complementary multi‐angle evaluation of XPI (Figure 2). The LPO and RPO projections portrayed the posterior right and left lung, respectively, without overlapping structures. The majority (*n* = 17) of the participants had XPI without spatial or global defects. One subject demonstrated globally low XPI despite technically adequate image acquisition (Figure 2c); the significance of this finding is unclear and warrants further investigation.

**FIGURE 3:**
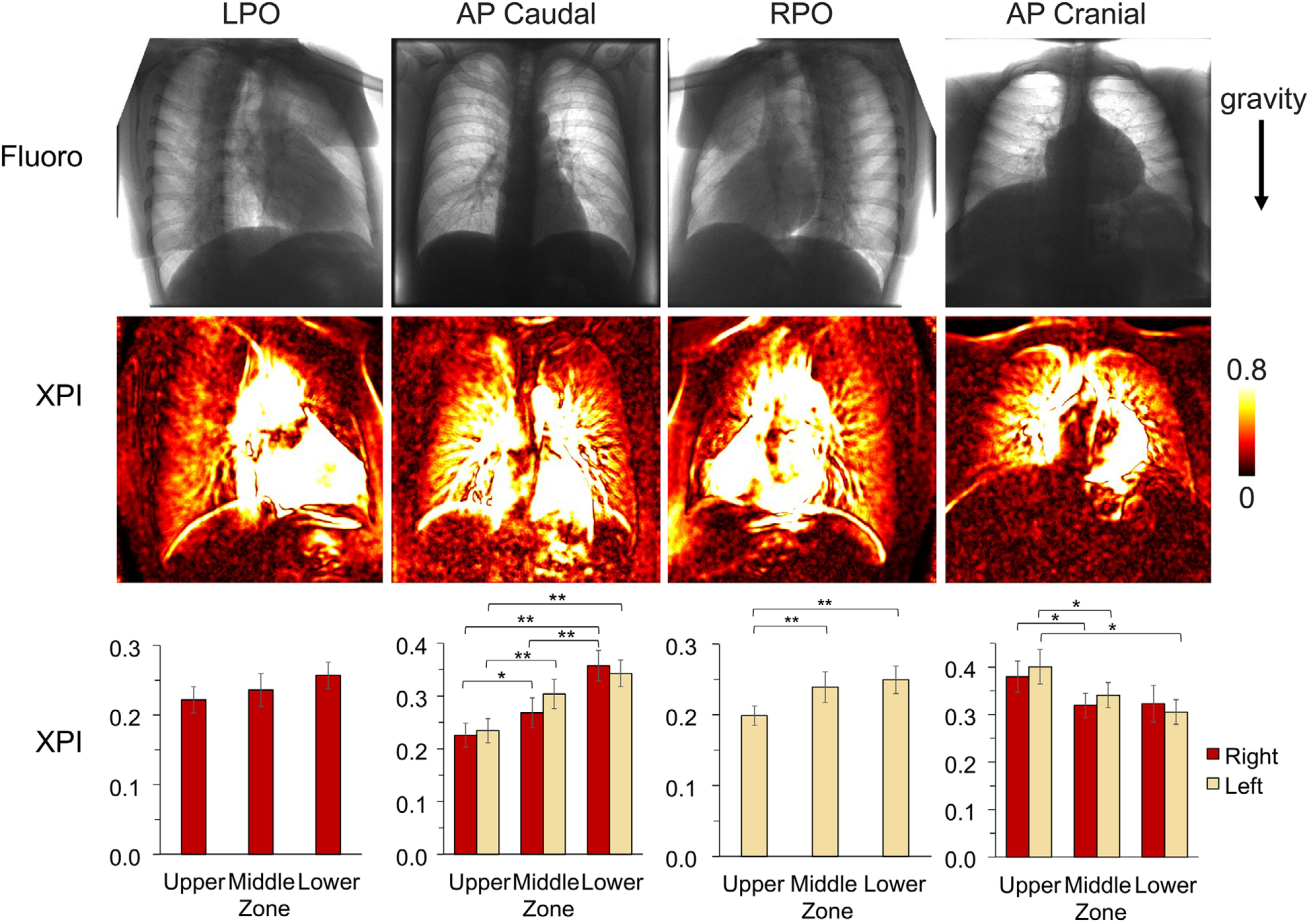
XPI distribution by projection angle and lung zone. Representative fluoroscopic images (top) and corresponding XPI maps (middle) are shown for LPO, AP caudal, RPO, and AP cranial projections. Bar graphs (bottom) summarize zonal XPI values (upper, middle, lower) for right (red) and left (tan) lungs. In oblique projections (LPO and RPO), higher XPI values are observed in the dependent posterior lung, demonstrating a significant cranial–caudal gradient (* *q* < 0.05, ** *q* < 0.01). The AP caudal projection best demonstrates this zonal dependence with relative lateral symmetry. In contrast, the AP cranial projection shows a reversal of the cranial–caudal gradient, likely reflecting superimposed mediastinal and cardiac structures in this view. No statistically significant right–left differences were detected using the LPO and RPO projections.

Figure 3 demonstrates how projection angle influences the visualization and quantitative assessment of regional XPI. The top and middle rows show representative radiographic projections paired with corresponding XPI images. The bottom row quantifies these XPI differences across upper, middle, and lower zones for both lungs. Significant cranial–caudal gradients in XPI were observed across multiple projection angles. Following Benjamini–Hochberg FDR adjustment, the upper zone measured significantly lower XPI values than the lower zones in the AP caudal and RPO views (*q* < 0.05), confirming the robustness of this physiologic pattern. The LPO projection demonstrated the same cranial–caudal trend as the other projections but did not meet the prespecified FDR significance threshold (*p* = 0.04, *q* = 0.06). The AP cranial view demonstrated an opposite pattern, with higher XPI values in the upper zones, likely due to projectional overlap from mediastinal structures in this view (see Figure 2a, AP Cranial). These findings suggest that gravity‐dependent regional physiology can strongly influence XPI in the upright seated position. There were no statistically significant laterality differences. XPI statistics and comparisons are provided in Table 1 and Supplemental 1.

**TABLE 1:** XPI values from the peripheral lung as a function of lung zone and projection angle.

	Right			Left		
	Upper	Middle	Lower	Upper	Middle	Lower
**LPO**	0.22 ± 0.02	0.24 ± 0.02	0.26 ± 0.02	NA	NA	NA
**AP Caudal**	0.23 ± 0.02	0.27 ± 0.03	0.36 ± 0.03	0.23 ± 0.02	0.30 ± 0.03	0.34 ± 0.03
**RPO**	NA	NA	NA	0.20 ± 0.01	0.24 ± 0.02	0.25 ± 0.02
**AP Cranial**	0.38 ± 0.03	0.32 ± 0.03	0.32 ± 0.04	0.40 ± 0.04	0.34 ± 0.03	0.30 ± 0.03

*Note*: *n* = 18, mean ± standard deviation.

**FIGURE 4:**
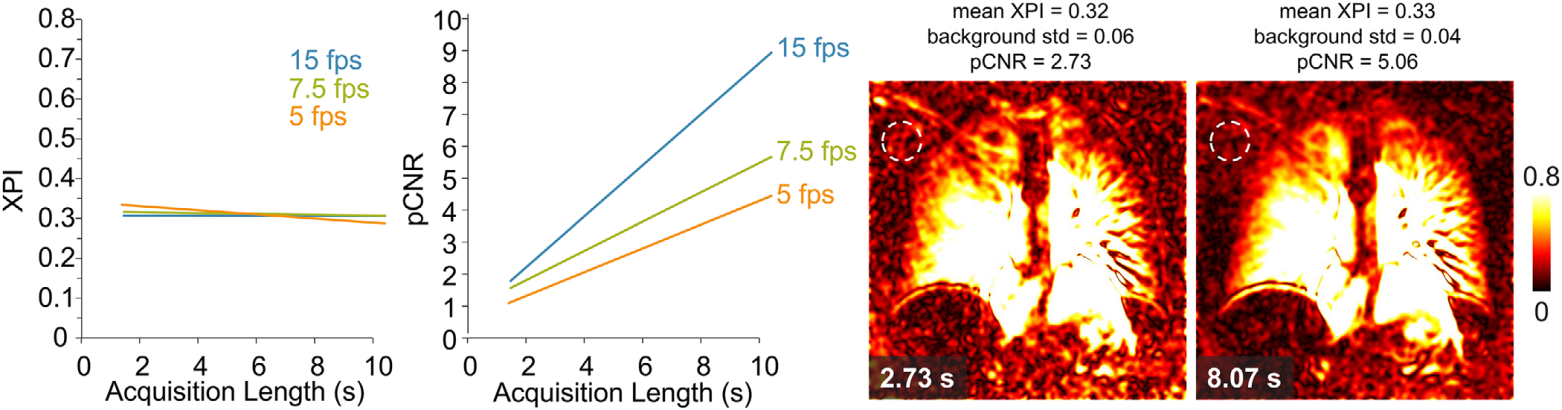
Retrospective temporal resampling demonstrates that mean XPI in the peripheral lung remains stable across acquisition lengths and frame rates (5, 7.5, and 15 fps), indicating robustness of the metric. In contrast, pCNR increases with acquisition length and higher frame rates, reflecting reduced noise and improved signal reliability with longer and faster acquisitions. Best‐fit linear regression line shown for both graphs. Representative images (right) illustrate these noise properties with varying acquisition duration.

XPI values remained robust with decreasing acquisition lengths regardless of frame rate (Figure 4). Reduced noise and improved signal reliability were observed with longer acquisitions and faster frame rates supported by changes in pCNR.

Entrance skin dose per tube and cumulative effective dose are summarized in Table 2. The estimated radiation dose based on standard participant sizes using 15 fps was calculated to be 0.55 mSv for an 8 s acquisition using four projection angles. The estimated dose lies between standard chest radiography (∼0.1 mSv) and chest CT angiography (∼2–5 mSv).

**TABLE 2:** Radiation dose estimates for each projection angle.

Tube (degrees)	SSD (cm)	Entrance Skin Exposure (mR)	Effective Dose (mSv)
LPO (55)	134.9	15.6	0.15
AP Caudal (22.5)	124.5	13.7	0.15
RPO (55)	135.5	12.0	0.13
AP Cranial (22.5)	135.9	9.5	0.12
Total	NA	NA	0.55

## 4 DISCUSSION

In this work, we evaluated XPI with healthy participants using a novel fluoroscopic system to establish preliminary reference values in four simultaneous projection angles. XPI reflects cyclic changes in x‐ray attenuation synchronized with the cardiac cycle, likely arising from a combination of pulmonary blood volume variation and vascular motion within the lung parenchyma, and is therefore interpreted here as a surrogate marker of regional perfusion. Maps generated with the system reproduced a well‐known gravity‐dependent upright perfusional physiology.^15^ The study of this phenomenon has traditionally only been observed in nuclear medicine studies,^16^ and this system offers new insight into the upright physiology of the lungs. Simultaneous multi‐angle views facilitated a three‐dimensional evaluation of the lungs with a single breath hold. As an internal control, there were no laterality differences.

The robust spectral processing of XPI allows for shorter scan times and radiation dose reductions with mild CNR penalty. While subjects in this study easily tolerated an 8 s breath hold, patients with severe pulmonary disease may not have the same capacity. Therefore, the results in this study demonstrate a shorter breath hold can produce XPI maps with acceptable CNR, especially when using a faster frame rate. Reducing the frame rate can also be an effective dose reduction strategy without sacrificing significant image quality.

One case demonstrating very low XPI was an outlier. Although this was an asymptomatic participant, they incidentally had low VO2 max, which is a measure of cardiorespiratory fitness. This finding raises the possibility that XPI may be sensitive to occult pulmonary vascular abnormalities. Additional studies involving cohorts with known disease will be investigated to test this theory.

A unique aspect of this system is the ability to image subjects in an upright seated position, enabling evaluation of gravity‐dependent perfusion patterns that are difficult to capture with conventional supine imaging modalities. The observed cranial–caudal gradient in XPI is consistent with classic descriptions of pulmonary perfusion distribution, as described by West,^15^, ^17^ in which dependent lung regions receive greater blood flow due to hydrostatic pressure effects. The ability to non‐invasively visualize this physiologic gradient using multi‐angle fluoroscopy provides a potential platform for studying dynamic vascular adaptation to postural change. Recent work by Mizoguchi et al. and Yamasaki et al. using DCR also explored perfusion changes with posture^9^, ^18^; however, those approaches inherently include substantial central lung signal, which is susceptible to projectional overlap and contributions from bulk cardiac motion. In contrast, the present study focused on the peripheral lung to mitigate these confounding factors and better isolate perfusion‐related signal. Building on this framework, a functional “stress test” paradigm could be envisioned in which XPI maps acquired in the supine position are compared to those obtained immediately after transition to an upright posture. The temporal dynamics and magnitude of perfusion redistribution may reflect pulmonary vascular compliance and overall cardiopulmonary fitness. While this concept remains speculative and was not directly evaluated in the present study, it highlights a potential application of this technique for probing pulmonary vascular physiology beyond static imaging.

There are some limitations of this work. This study did not have a visual comparison with a gold standard, but this was done in a prior study using digital subtraction angiography.^13^ Reference values for XPI have not been previously established, but the lateral symmetry and cranial‐caudal differences reflect well‐known physiology. No standardized quantitative metric currently exists for fluoroscopy‐derived regional lung perfusion assessment. XPI is overestimated when projectional overlap occurs, causing superimposition of multiple vascular structures adding to the x‐ray attenuation. This is only the case centrally with large vascular structures such as the heart, aorta, and main pulmonary arteries. Therefore, XPI measurements were taken from the periphery to avoid this bias. Reproducibility of segmentation was not formally assessed and represents a limitation of this study, but could be replaced by an automated procedure for scalability. This study employed a CNR measurement reflecting the image quality and suppression of background signal and background noise. This metric is important for detecting low XPI areas like perfusion defects. In addition, the lung was divided into three vertical zones that do not correspond to anatomic lobes. Therefore, care should be taken if comparing to other 3D perfusion techniques such as CTA or pulmonary scintigraphy. The sample size and predominantly male cohort limits generalizability of the reported reference values. Although the Benjamini–Hochberg procedure was used to control the false discovery rate across multiple planned comparisons, statistically significant findings should still be interpreted with appropriate caution, as false discoveries may occur despite FDR correction. Confirmation in larger cohorts will be important to establish the reproducibility of these regional differences. Finally, the radiation dose estimate is a reference point not specific to any one participant's study but rather based on a test acquisition to a phantom. Individual doses to participants would vary based on body habitus and x‐ray technique. A more complete radiation dose estimate and comparison to other modalities and reference points will be conducted in future work on this data set.

## 5 CONCLUSION

This pilot study establishes baseline XPI measurements, a surrogate for lung perfusion, and demonstrates feasibility of multi‐angle fluoroscopic lung perfusion imaging within a single breath hold.

## CONFLICT OF INTEREST STATEMENT

Authors AF, GTM, and CRH are employees of 4DMedical, which developed the imaging device evaluated in this study. The remaining authors declare no conflicts of interest.

## Supporting information



Supporting information: mp70666‐sup‐0001‐Table S1.docx
