# Supplementary material for: Multi‐angle fluoroscopic lung perfusion imaging using x‐ray pulsatility index: A pilot study in healthy subjects
Source: Med Phys. 2026 Sep 2;53(9):e70666. doi: 10.1002/mp.70666 (PMC13535757; doi:10.1002/mp.70666)
Supplement: Supplementary file 1 [file MP-53-0-s001.docx]

**S1.** Pairwise comparisons of lobar peripheral XPI across projection angles.

| **Projection** | **Laterality** | **Comparison** | **p-value** | **q-value** | **Significant** |
| --- | --- | --- | --- | --- | --- |
| LPO | Right | Upper vs Lower | 0.037 | 0.0714 | No |
| LPO | Right | Middle vs Lower | 0.150 | 0.2106 | No |
| LPO | Right | Upper vs Middle | 0.289 | 0.3567 | No |
| AP Caudal | Right | Upper vs Lower | 0.000 | 0.0012 | ** |
| AP Caudal | Right | Middle vs Lower | 0.002 | 0.0068 | ** |
| AP Caudal | Right | Upper vs Middle | 0.009 | 0.0180 | * |
| AP Caudal | Left | Upper vs Lower | 0.000 | 0.0012 | ** |
| AP Caudal | Left | Middle vs Lower | 0.041 | 0.0724 | No |
| AP Caudal | Left | Upper vs Middle | 0.000 | 0.0012 | ** |
| RPO | Left | Upper vs Lower | 0.000 | 0.0023 | ** |
| RPO | Left | Middle vs Lower | 0.439 | 0.5118 | No |
| RPO | Left | Upper vs Middle | 0.002 | 0.0079 | ** |
| AP Cranial | Right | Upper vs Lower | 0.238 | 0.3125 | No |
| AP Cranial | Right | Middle vs Lower | 0.914 | 0.9141 | No |
| AP Cranial | Right | Upper vs Middle | 0.005 | 0.0128 | * |
| AP Cranial | Left | Upper vs Lower | 0.005 | 0.0128 | * |
| AP Cranial | Left | Middle vs Lower | 0.118 | 0.1775 | No |
| AP Cranial | Left | Upper vs Middle | 0.005 | 0.0128 | * |
| LPO/RPO | Both | Upper vs Upper | 0.054 | 0.0865 | No |
| LPO/RPO | Both | Middle vs Middle | 0.804 | 0.8441 | No |
| LPO/RPO | Both | Lower vs Lower | 0.686 | 0.7581 | No |

*q < 0.05; **q < 0.01
